# Perceived Menstrual Irregularities and Premenstrual Syndrome in Relation to Insomnia: Evidence from a Cohort of Student Nurses

**DOI:** 10.3390/jcm14217470

**Published:** 2025-10-22

**Authors:** Anastasiia Dimlievych, Grażyna Dębska, Joanna Grzesik-Gąsior, Anna Merklinger-Gruchala

**Affiliations:** 1 Faculty of Health Sciences, Medical College, Andrzej Frycz Modrzewski Krakow University, Herlinga-Grudzińskiego 1, 30-705 Kraków, Poland; dimlievych@gmail.com (A.D.); gdebska@uafm.edu.pl (G.D.); 2 State University of Applied Sciences in Krosno, Rynek 1, 38-400 Krosno, Poland; joanna.grzesik-gasior@pans.krosno.pl

**Keywords:** insomnia, menstrual cycle, premenstrual syndrome, sleep quality, menstrual irregularities

## Abstract

**Background/Objectives**: Sleep disorders, particularly insomnia, are increasingly recognized as key determinants of mental health. Disturbances in sleep architecture may exacerbate hormonal dysregulation, contributing to menstrual cycle irregularities and premenstrual syndrome (PMS). The study investigate the relationship between insomnia symptoms, menstrual problems, and PMS among nursing students. **Methods**: The cross-sectional study was conducted using a web-based survey (CAWI) among 72 female graduate nursing students. The questionnaire included questions about menstrual history, the presence of menstrual disorders, PMS symptoms, and lifestyle and body mass index (BMI). Insomnia was assessed using the Polish version of the Athens Insomnia Scale (AIS), taking ≥8 as the cutoff point. Logistic regression analysis with confounding variables was performed. **Results**: 70% of participants had PMS symptoms, 19.5% had irregular menstrual cycles, and 86.5% reported problems with menstrual bleeding. The mean AIS score was 10.1 (SD = 4.05). Women with insomnia were almost 4 times more likely to experience PMS symptoms (OR = 3.93; 95% CI 1.14–13.59), more than 7 times more likely to experience bleeding problems (OR = 7.56; 95% CI: 1.51–37.97), and each additional AIS score increased the risk of cycle irregularity by 24% (OR = 1.24, 95% CI 1.01–1.50). **Conclusions**: The findings indicate a significant association between insomnia symptoms, menstrual disturbances, and PMS, underscoring the complex links between sleep, reproductive, and mental health. Preventive interventions, particularly sleep hygiene education, may serve as an effective strategy to support women’s overall health and well-being.

## 1. Introduction

Sleep problems represent a growing public health concern that affects a substantial proportion of the global population. Among students in medical and health-related disciplines, sleep disturbances have been reported at particularly students engaged in shift work [1]. A recent meta-analysis encompassing 109 studies worldwide reported that 57% of medical students worldwide experienced poor sleep quality, with 33% suffering from excessive daytime sleepiness [2]. These findings underscore the scale of the problem and highlight the vulnerability of young adults pursuing demanding health-related careers. Nursing students, particularly female students, face a unique set of challenges that can significantly impact their physical and mental health. These challenges include high levels of stress, irregular schedules, demanding workloads, and lack of life-work balance all of which can contribute to the development of various health issues, such as circadian rhythm disturbances and insomnia [1]. Sleep disorders can consequently lead not only to overweight or obesity [3], but have also been associated with menstrual health problems, including premenstrual syndrome (PMS) and other cycle disturbances. A study tracking newly employed nurses over 12 months found that insomnia was linked to a 2-fold increase in the odds of developing menstrual cycle irregularity [4]. Sleep quality also appears to fluctuate across different phases of the menstrual cycle, with higher sleep efficiency and shorter sleep latency during the follicular phase compared to the menstruation and luteal phases [5]. Sleep disturbances (insomnia symptoms) was found to be associated with irregular menstrual cycles [6]. Contrary, longer sleep duration may diminish the prevalence of menstrual cycle irregularity [7]. Furthermore, community-based studies have demonstrated the associations between sleep disturbances (short sleep duration and poor sleep quality) not only with greater menstrual cycle irregularity but also with heavier menstrual bleeding [8]. These findings indicate a bidirectional relationship between sleep and menstrual function, where sleep not only responds to hormonal fluctuations but may also influence the severity and regularity of menstrual symptoms.

Poor sleep quality may also be associated with premenstrual syndrome (PMS), with sleep changes being a strong predictor of PMS severity, what was found among nursing and medical students [9]. Clinical nurses may have a particularly high prevalence of PMS [10], which was found to significantly correlate with sleep quality [10]. Further, sleep quality may play an important intermediary role between occupational stress and PMS among this group of women, what was noted among nurses in China. Research on newly graduated nurses has demonstrated that the onset of shift work can exacerbate PMS symptoms, with persistent effects over time [11]. Despite the growing body of evidence connecting sleep and reproductive health, research on this topic remains limited in populations that are simultaneously exposed to academic and clinical stress—such as student nurses. Moreover, few studies have examined multiple menstrual outcomes (cycle regularity, bleeding disturbances, and PMS symptoms) in relation to insomnia within a single analytical model. This gap in the literature is critical, considering the unique vulnerability of nursing students to both sleep disruption and menstrual disorders due to the overlapping demands of education and early professional experience.

In light of this evidence, the present study aimed to examine whether symptoms of insomnia, as measured by the Athens Insomnia Scale (AIS), are associated with menstrual problems among nursing students. Based on the previous findings [12], we hypothesized that insomnia contributes to the occurrence of menstrual problems, such as menstrual irregularities, scanty or heavy menstrual bleeding and the occurrence of symptoms related to premenstrual syndrome.

## 2. Materials and Methods

### 2.1. Study Design and Participants

A cross-sectional design was applied using a self-administered, online questionnaire. The study was conducted using a diagnostic survey based on the CAWI method (Computer-Assisted Web Interview)**.** Data were collected between October 2023 to January 2024 using the digitalized research tool (Google forms), consisted the questions about reproductive history (age of menarche), occurrence of menstrual problems, i.e., irregular menstrual cycles, problems with menstrual bleeding, and symptoms related to premenstrual syndrome, gender, age, length of service in the profession (professional experience), as well as the type of work performed (shift, single shift—only during the day, single shift—only at night, no work). It also included questions on anthropometric indicators, i.e., body weight and height of the participants, were also included, on the basis of which the BMI (Body Mass Index) was calculated. The Athens Insomnia Scale (AIS), in a validated Polish version [13] was used to measure insomnia symptoms during the last 3 months, based on ICD-10 criteria. High sensitivity (93%) and specificity of the scale (85%) were demonstrated. Consent to use the validated version of the Athens Insomnia Scale (AIS) in the Polish language version was obtained.

The study population consisted of first and second-year master’s female nursing students, aged 22–45 years, from a one of Krakow’s universities in Poland. Inclusion criteria were: current menstruation, no diagnosis of gynecological or psychiatric disorders, and voluntary participation. Exclusion criteria included pregnancy, lactation, menopause, endocrine disorders (e.g., PCOS, thyroid dysfunction) and diagnosed psychiatric or eating disorders.

### 2.2. Ethical Considerations

The research project and tool were approved by the University Bioethics Committee No. KBKA/55/O/2023 (19 October 2023). Each student gave their consent to participate in the study and was asked to read the detailed information about the purpose and nature of the study during which they could withdraw their participation at any time. No sensitive or personally identifiable data were collected in this anonymous study.

### 2.3. Data Management and Statistical Procedure

According to the results of validation studies, a score of ≥8 points on the AIS was considered to indicate the presence of insomnia problems [13]. Menstrual problems were assessed in four aspects based on the participants’ self-reported experiences: (1) menstrual cycle irregularities—defined as polymenorrhoea (cycles shorter than 21 days) and oligomenorrhoea (cycles longer than 35 days); (2) abnormal bleeding volume—including hypomenorrhoea, hypermenorrhoea, and menorrhagia; (3) dysmenorrhea—defined as painful menstruation; and (4) the presence of symptoms suggestive of premenstrual syndrome, along with their impact on daily social functioning and interpersonal relationships. This criteria was adopted according to American College of Obstetricians and Gynecologists (ACOG) recommendations and ICD-10 for PMS diagnoses. PMS was operationally defined by the following: (1) the presence of one or more psychological or physical symptoms occurring in the period from 5 days before monthly bleeding and disappear up to 4 days after the onset of menstruation; (2) the absence of symptoms in the follicular phase of the menstrual cycle; (3) symptoms occur to a moderate or severe degree, which impairs the woman’s social and family functioning and causes significant physical and/or psychological discomfort, strong enough to seek help from a specialist; (4) symptoms occur during most menstrual cycles and must be confirmed prospectively in an observation diary kept for a minimum of two months; (5) symptoms occurring not may be an exacerbation of an existing mental disorder or other illnesses. PMS criteria were operationalized by asking whether each of the following 12 symptoms occurred during the previous 12 months (feeling of “loss of control”, depressed mood, anxiety/tension, mood lability, irritability, social friction, decreased concentration, appetite changes, sleep problems, bloating, headache, breast swelling, and tenderness), and to what extent each symptom was bothersome, rated on a 5-point Likert scale ranging from “not bothersome at all” to “very bothersome”. To identify participants with symptom patterns suggestive of PMS, two additional questions were included. First, respondents were asked whether the listed symptoms appeared or worsened during the luteal phase (premenstrual phase) and subsided or improved at the beginning of the follicular phase, or whether they were unrelated to the menstrual cycle phase. Second, participants were asked whether these symptoms interfered with their functioning at work or university and/or with daily social activities and interpersonal relationships, with response options: yes, rather yes, rather no, and no. Participants who reported cyclical symptom patterns and at least some interference with functioning were classified as suggesting the presence of PMS.

### 2.4. Statistical Analysis

Descriptive statistics were used to describe the characteristics of the studied group. The PMS groups (yes/no) were compared by using Mann–Whitney U test and χ^2^ test with Yates’ correction. The simple and multiple logistic regression analysis were used to assess the effect of insomnia (both as the categorical and continuous variable) on PMS occurrence, menstrual cycle irregularity and bleeding problems. The *p*-value of <0.05 was considered statistically significant. Analysis were performed with Statistica ver. 14.1.0.4. TIBCO Software Inc. (San Ramon, CA, USA).

## 3. Results

### 3.1. Participants’ Characteristics

During the study period, *N* = 49 first-year and *N* = 62 second-year nursing students collected the questionnaires. Out of which women who declared amenorrhea due to entering the perimenopausal period (*n* = 5), or due to pregnancy or breastfeeding) (*n* = 1) and woman due to extremely high BMI (>1.5 IQR) (*n* = 1) were excluded from the study. The final size of the study group was: *N* = 72 participants. The participants characteristics according to the PMS occurrence was presented in Table 1.

### 3.2. Menstrual Problems Frequency

**Figure 1:**
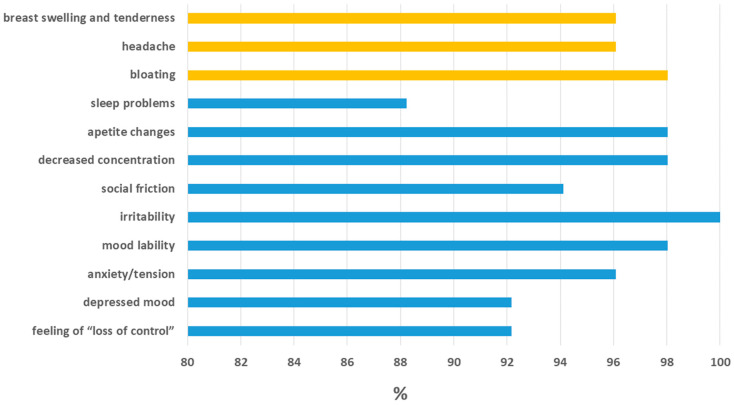
Prevalence of PMS symptoms among female students (*n* = 72). Physical symptoms (yellow bars), emotional/behavioral symptoms (blue bars).

Among the 72 female students of reproductive age (range: 21.0–48.0 years, with a median age of 23.5), *n* = 51 respondents declared symptoms suggesting the occurrence of PMS (70.1%), i.e., they declared that at least one of the symptoms listed in the survey occurred, (Figure 1) and they appear or worsen during the luteal phase of the cycle and disappear or milder at the beginning of the follicular phase. Among the *N* = 72 women, *n* = 58 had a regular menstrual cycle (80.6%), i.e., a cycle that lasts from 21 to 35 days, whilst *n* = 14 women indicated a lack of regularity of their menstrual cycle (19.47%). Among the respondents, the greatest number of women indicated feeling pain during bleeding (*n* = 44, 59.46%). Very heavy bleeding was reported by *n* = 27 women (36.49%), whilst *n* = 7 women indicated very scanty bleeding (9.46%). Bleeding lasting more than 7 days was indicated by 3 respondents (4.05%). In total, bleeding problems, i.e., the occurrence of at least one of the above symptoms, were declared by *n* = 64 women examined (86.49%).

### 3.3. Insomnia Frequency

The mean AIS score among the participants was 10.1 ± 4.1. The analysis of the PMS groups in relation to the AIS items revealed that participants with PMS more often reported insufficient sleep duration (1.59 Vs. 1.29; *p* = 0.03), poorer well-being during the day (1.57 Vs. 1.00; *p* = 0.01), impaired daytime functioning (1.45 Vs. 0.62; *p* < 0.001), greater daytime sleepiness (1.88 Vs. 1.38; *p* < 0.001), and earlier than desired final awakening (0.47 Vs. 1.00; *p* = 0.02). No significant differences were observed in the remaining AIS items. The total AIS score was higher in the PMS group (10.59 Vs. 9.00), but this difference did not reach a statistical significance (*p* = 0.13) (Table 2).

### 3.4. Association Between Insomnia and Menstrual Problems

A statistically significant association was observed between insomnia and the occurrence of PMS symptoms, cycle irregularity, and bleeding problems among respondents, after adjusting for potential confounders (work experience, night-shift work, BMI, age, and age at menarche) (Table 3). Women with insomnia (AIS ≥ 8 Points Vs. <8 points) were almost four times more likely to report PMS symptoms (OR = 3.93, 95% CI: 1.14–13.59). Each additional AIS score was associated with a 15% increase in the likelihood of PMS (*p* = 0.06) and a 24% increase in the likelihood of cycle irregularity (OR = 1.24, 95% CI: 1.01–1.50, *p* = 0.04). Students with insomnia (AIS ≥ 8 Points Vs. <8 points) were over seven times more likely to report bleeding problems (OR = 7.56, 95% CI: 1.51–37.97, *p* = 0.01). Each additional AIS score increased the odds of bleeding problems by 51% (OR = 1.51, 95% CI: 1.13–2.00, *p* < 0.001). Given the relatively small sample size and wide confidence intervals, these estimates should be interpreted with caution and considered exploratory.

## 4. Discussion

Sleep disorders may play an important role in the development of menstrual cycle disorders. Insomnia may contribute to problems such as irregular menstrual cycles, scanty or heavy menstrual bleeding, and premenstrual syndrome (PMS) symptoms [5,6]. In the present study, we attempted to verify whether insomnia symptoms are associated with menstrual problems in a group of women in a phase of intensive professional and educational development.

We found that women with insomnia (AIS ≥ 8) showed higher odds of reporting PMS, with each additional AIS point associated with an approximately 15% increase in the likelihood of PMS. Experiencing PMS was also linked to a less favorable sleep profile, including shorter sleep duration, greater daytime sleepiness, and reduced daytime well-being and functioning. Women with PMS more often reported an earlier than desired final awakening, suggesting that sleep continuity and restorative quality may be particularly affected in this group. Although the total AIS score was higher among women with PMS compared to those without, this difference did not reach statistical significance, which may reflect the limited sample size.

Women with insomnia (AIS ≥ 8) also demonstrated higher odds of reporting cycle irregularity (by about 24%) and bleeding problems (over sevenfold higher odds), with each AIS point associated with a 51% increase in the likelihood of bleeding problems. These results were consistent with studies on sleep problems among women in various populations, particularly in East Asia. For instance, a study among female university students in China found that insomnia symptoms were significantly associated with menstrual problems such as prolonged menstrual flow (≥7 days), period pain, irregular cycles, and premenstrual syndrome [14]. Likewise, a study from Taiwan reported significant associations between poor sleep quality, fatigue, and increased rates of menstrual irregularities and bleeding problems [8]. The results of our study are also consistent with the findings from newly employed nurses in South Korea, indicating that insomnia was associated with a 2.05-fold increase in the risk of developing menstrual cycle irregularities over 12 months and a 3.05-fold increase in the frequency of menstrual cycle irregularities [4]. Another study on women of reproductive age, but with chronic insomnia disorder found high percentages of hypomenorrhea, dysmenorrhea, and premenstrual syndrome [15]. Severe sleep disturbance in the past month was also associated with reduced ovarian reserve, suggesting that sleep disorders may affect both menstrual health and reproductive capacity [15].

While considering the association between sleep quality or insomnia and menstrual cycle characteristics, it is important to emphasize the high relevance of psychosocial stress and mental health problems [16]. Premenstrual Syndrome is increasingly recognized not only as a physiological condition but also as a psychological issue [17]. It has been demonstrated that PMS significantly correlates with sleep disturbances, showing co-occurrence of insomnia, poor sleep quality, and excessive daytime sleepiness, particularly during the luteal phase of the menstrual cycle [18]. Women suffering from the more severe form of PMS, i.e., Premenstrual Dysphoric Disorder (PMDD), experience even more pronounced symptoms of insomnia and fatigue, especially in the luteal phase, which markedly affects their daily functioning. These effects may be modulated by disruptions in circadian rhythms and melatonin secretion [19]. Therefore, the relationship between insomnia and menstrual issues can be regarded as bidirectional. On one hand, chronic insomnia can intensify menstrual irregularities by disrupting the hypothalamic-pituitary-gonadal axis and elevating the stress response [20,21]. On the other hand, hormonal variations in ovarian steroids during the menstrual cycle, along with PMS symptoms, can reciprocally impair sleep initiation and maintenance [22]. This interplay is mediated by the widespread presence of estrogen and progesterone receptors in the central nervous system, particularly in regions critical for sleep regulation such as the basal forebrain, hypothalamus, dorsal raphe nucleus, and locus coeruleus [22].

When discussing the results of this study, it is important to consider certain limitations. As this study was cross-sectional, causal relationships cannot be firmly established. Insomnia and menstrual symptoms may mutually reinforce each other via stress or hormonal dysregulation pathways, a possibility that future longitudinal work could further explore. Secondly, the omission of information regarding hormonal contraception status, recent discontinuation of hormonal contraceptives, caffeine consumption, herbal therapy, nutraceuticals, dietary supplements, or perceived stress represents a significant limitation. These factors may significantly influence both sleep and menstrual regularity. Moreover, the identification of PMS was based on participants’ self-assessment using established diagnostic criteria from the American College of Obstetricians and Gynecologists (ACOG) and ICD-10, rather than clinical diagnosis. However, this approach is consistent with other population-based studies and reflects real-world symptom perception among women. The sample size (*N* = 72), while relatively modest, was supported by post hoc power analysis, which indicated that with a significance level of α = 0.05, and detected effect size, the statistical power reached 85%. This suggests that the sample size was sufficient to reliably detect an association between insomnia and menstrual problems. However, given this modest sample size and wide confidence intervals (e.g., OR = 7.56 for bleeding problems), the possibility of a type I error cannot be ruled out, and the observed associations should therefore be interpreted with caution.

Furthermore, the study focused on a working nursing students balancing both academic and professional demands, offering real-world insight into a group at particular risk of health deterioration due to chronic stress and sleep deprivation. The findings highlight a significant issue of sleep disturbances in this population, likely stemming from the need to balance multiple roles, resulting in a heavy workload and reduced time for rest. Importantly, the study indicated that women of reproductive age within this group may be particularly vulnerable to menstrual disturbances associated with insomnia. Employer support strategies, such as optimizing shift schedules and reducing the frequency of consecutive night shifts, may help to reduce the occurrence of sleep problems and insomnia symptoms. Since working three or more consecutive night shifts increases the risk of insomnia by 2.33 times (research in South Korea [23]), it is worth considering reducing the intensity of night shifts, which could reduce the negative effects of sleep problems on reproductive health. Additionally, the implementation of sleep hygiene training for working student nurses may improve sleep quality and help reduce the symptoms of insomnia. Future prospective studies and intervention-based research could help identify effective strategies for mitigating sleep-related menstrual disturbances and enhancing overall well-being among healthcare trainees.

## 5. Conclusions

Insomnia symptoms were significantly associated with menstrual disorders among female nursing students, including both cycle irregularity, bleeding problems and premenstrual syndrome symptoms. The results support the hypothesis that insomnia may contribute to various menstrual problems in women of reproductive age. Occupational factors, such as shift work and the stress of combining educational and occupational responsibilities, may provide a significant background for the exacerbation of insomnia symptoms and, indirectly, menstrual disorders. Previous research by Rapkin and Winer [24] demonstrated that PMS significantly affects women’s quality of life, primarily through psychological symptoms that interfere with daily functioning, including academic performance and social engagement. This highlights the broader psychosocial impact of menstrual disorders in women. Therefore, a holistic approach to women’s health is essential, especially one that addresses both sleep quality and reproductive health. The introduction of targeted educational, and organizational interventions could substantially improve the quality of life and help reduce the negative health outcomes in this population.

## Figures and Tables

**Table 1:** Characteristics of the participants according to the PMS symptoms occurrence.

Characteristics			PMS		Test	*p*-Value
			Yes	No		
Cycle regularity	yes	*n* (%)	40 (78.4)	18 (85.7)	Chi^2^ = 0.50, df = 1	0.48
	no	*n* (%)	11 (21.6)	3 (14.3)		
Age	<25	*n* (%)	30 (58.8)	13 (61.9)	Chi^2^ = 0.06, df = 1	0.81
	≥25	*n* (%)	21 (41.2)	8 (38.1)		
Parity	no	*n* (%)	38 (74.5)	15 (71.4)	Chi^2^ = 0.07, df = 1	0.79
	yes	*n* (%)	13 (25.5)	6 (28.6)		
Night shift work	no	*n* (%)	14 (27.5)	3 (14.3)	Chi^2^ = 1.42, df = 1	0.23
	yes	*n* (%)	37 (72.6)	18 (85.7)		
Professional experience	no or <1 year	*n* (%)	22 (43.1)	5 (23.8)	Chi^2^ = 2.37, df = 1	0.30
	1–5 years	*n* (%)	16 (31.4)	9 (42.7)		
	>5 years	*n* (%)	13 (25.5)	7 (33.3)		
			PMS		Z	*p*
			yes	no		
Body mass	[kg]	*n*	51	21	0.10	0.92
		Median (Q1–Q3)	62.0 (58.0–68.0)	62.0 (57.0–68.0)		
Height	[cm]	*n*	51	21	2.43	0.02
		Median (Q1–Q3)	166.0 (164.0–170)	164.0 (160.0–167.0)		
BMI	[kg/m^2^]	*n*	51	21	−0.95	0.34
		Median (Q1–Q3)	22.2 (21.0–24.0)	23.1 (21.5–26.5)		
Menarcheal age	[years]	*n*	50	21	−0.08	0.94
		Median (Q1–Q3)	13.0 (12.0–14.0)	13.0 (12.0–14.0)		
AIS	[total score]	*n*	51	21	1.35	0.18
		Median (Q1–Q3)	11.0 (8.0–13.0)	8.0 (5.0–13.0)		

**Table 2:** Insomnia problem frequency (AIS items) according to PMS groups.

Item Score	*N*	PMS (Yes)		*N*	PMS (No)		Test	*p*-Value
		Mean (SD)	Me (Q1–Q3)		Mean (SD)	Me (Q1–Q3)		
Sleep induction [0–3]	51	1.24 (0.79)	1.00 (1.0–2.0)	21	1.24 (1.0)	1.00 (0.0–2.0)	Z = 0.00	1.00
Awakenings during the night [0–3]	51	0.90 (0.73)	1.00 (0.0–1.0)	21	1.33 (0.9)	1.00 (1.0–2.0)	Z = −1.94	0.05
Final awakening earlier than desired [0–3]	51	0.47 (0.73)	0.00 (0.0–1.0)	21	1.00 (1.0)	1.00 (0.0–2.0)	Z = −2.31	0.02
Total sleep duration [0–3]	51	1.59 (0.78)	2.00 (1.0–2.0)	21	1.29 (0.6)	1.00 (1.0–2.0)	Z = 2.14	0.03
Overall quality of sleep [0–3]	51	1.49 (0.67)	2.00 (1.0–2.0)	21	1.14 (0.9)	1.00 (1.0–2.0)	Z = 1.74	0.08
Sense of well-being during the day [0–3]	51	1.57 (0.81)	2.00 (1.0–2.0)	21	1.00 (0.7)	1.00 (1.0–1.0)	Z = 2.79	0.01
Functioning during the day [0–3]	51	1.45 (0.73)	2.00 (1.0–2.0)	21	0.62 (0.5)	1.00 (0.0–1.0)	Z = 4.27	0.00
Sleepiness during the day [0–3]	51	1.88 (0.59)	2.00 (2.0–2.0)	21	1.38 (0.6)	1.00 (1.0–2.0)	Z = 3.00	0.00
AIS [total score]	51	10.59 (3.69)	11.00 (8.0–13.0)	21	9.00 (4.7)	8.00 (5.0–13.0)	t = 1.5, df = 70	0.13

**Table 3:** Crude and adjusted effect of insomnia on the menstrual problems among respondents.

Outcome Measure	Insomnia Effect	Crude	*p*-Value	Adjusted	*p*-Value	AUC (SE)	Hosmer Lemeshow Test (HL)
		OR (95% CI)		OR (95% CI)			
PMS symptoms occurrence	AIS ≥ 8 points	2.44 (0.83–7.18)	0.11	3.93 (1.14–13.59)	0.03	0.74 (0.07)	HL = 4.1; *p* = 0.85
	AIS total score	1.11 (0.97–1.26)	0.13	1.15 (1.00–1.32)	0.06	0.76 (0.07)	H-L = 9.4; *p* = 0.30
Cycle irregularity	AIS ≥ 8 points	1.95 (0.49–7.74)	0.34	3.00 (0.55–16.37)	0.20	0.73 (0.07)	HL = 8.5; *p* = 0.39
	AIS total score	1.10 (0.95–1.27)	0.19	1.24 (1.01–1.50)	0.04	0.77 (0.07)	H-L = 3.2; *p* = 0.92
Bleeding problems	AIS ≥ 8 points	6.00 (1.34–26.81)	0.02	7.56 (1.51–37-97)	0.01	0.76 (0.10)	H-L = 8.1; *p* = 0.42
	AIS total score	1.48 (1.15–1.91)	<0.001	1.51 (1.13–2.00)	<0.001	0.87 (0.08)	H-L = 15.1; *p* = 0.06

## Data Availability

The raw data supporting the conclusions of this article will be made available by the authors on request.

## Abbreviations

The following abbreviations are used in this manuscript:

ACOG	American College of Obstetricians and Gynecologists
AIS	Athens Insomnia Scale
BMI	Body Mass Index
CAWI	Computer-Assisted Web Interview (web-based survey)
PCOS	Polycystic Ovary Syndrome
PMDD	Premenstrual Dysphoric Disorder
PMS	Premenstrual Syndrome

## References

[1] Thomas C.M., McIntosh C.E., Lamar R.A., Allen R.L. (2016). Sleep deprivation in nursing students: The negative impact for quality and safety. J. Nurs. Educ. Pract..

[2] Binjabr M.A., Alalawi I.S., Alzahrani R.A., Albalawi O.S., Hamzah R.H., Ibrahim Y.S., Buali F., Husni M., BaHammam A.S., Vitiello M.V. (2023). The worldwide prevalence of sleep problems among medical students by problem, country, and COVID-19 status: A systematic review, meta-analysis, and meta-regression of 109 studies involving 59,427 participants. Curr. Sleep Med. Rep..

[3] Huang H., Yu T., Liu C., Yang J., Yu J. (2024). Poor sleep quality and overweight/obesity in healthcare professionals: A cross-sectional study. Front. Public Health.

[4] Kang W., Jang K.-H., Lim H.-M., Ahn J.-S., Park W.-J. (2019). The menstrual cycle associated with insomnia in newly employed nurses performing shift work: A 12-month follow-up study. Int. Arch. Occup. Environ. Health.

[5] Ishikura I., Fernandes G., Hachul H., Tufik S., Andersen M. (2023). 0742 Is sleep altered during the menstrual cycle? A polysomnography study from EPISONO. Sleep.

[6] Liu X., Chen H., Liu Z.-Z., Fan F., Jia C.-X. (2017). Early menarche and menstrual problems are associated with sleep disturbance in a large sample of Chinese adolescent girls. Sleep.

[7] Nam G.E., Han K., Lee G. (2017). Association between sleep duration and menstrual cycle irregularity in Korean female adolescents. Sleep Med..

[8] Kennedy K.E.R., Onyeonwu C., Nowakowski S., Hale L., Branas C.C., Killgore W.D.S., Wills C.C.A., Grandner M.A. (2022). Menstrual regularity and bleeding is associated with sleep duration, sleep quality and fatigue in a community sample. J. Sleep Res..

[9] Erbil N., Yücesoy H. (2022). Relationship between premenstrual syndrome and sleep quality among nursing and medical students. Perspect. Psychiatr. Care.

[10] Wang X., Ge Y., Liu Y., Hu W., Wang Y., Yu S. (2024). The association between occupational stress, sleep quality and premenstrual syndrome among clinical nurses. BMC Nurs..

[11] Huh I., Choi-Kwon S., Ki J., Kim S., Baek J. (2024). Premenstrual Symptoms Risk Factors Among Newly Graduated Nurses in Shift Work: A Prospective Longitudinal Study. Asian Nurs. Res..

[12] Lin P.-C., Ko C.-H., Lin Y.-J., Yen J.-Y. (2021). Insomnia, Inattention and Fatigue Symptoms of Women with Premenstrual Dysphoric Disorder. Int. J. Environ. Res. Public Health.

[13] Fornal-Pawłowska M., Wołończyk-Gmaj D., Szelenberger W. (2011). Walidacja Ateńskiej Skali Bezsenności. Psychiatr. Pol..

[14] Xing X., Xue P., Li S.X., Zhou J., Tang X. (2020). Sleep disturbance is associated with an increased risk of menstrual problems in female Chinese university students. Sleep Breath..

[15] Gong M., Gao Y., Wang Z., Lu F., Dong H. (2024). The impact of chronic insomnia disorder on menstruation and ovarian reserve in childbearing-age women: A cross-sectional study. Clin. Exp. Reprod. Med..

[16] Yu M., Han K., Nam G.E. (2017). The association between mental health problems and menstrual cycle irregularity among adolescent Korean girls. J. Affect. Disord..

[17] Takeda T. (2023). Premenstrual disorders: Premenstrual syndrome and premenstrual dysphoric disorder. J. Obstet. Gynaecol. Res..

[18] Delray K., Lewis G., Hayes J.F. (2025). Tracking mood symptoms across the menstrual cycle in women with depression using ecological momentary assessment and heart rate variability. BMJ Ment. Health.

[19] Yin W., Zhang J., Guo Y., Wu Z., Diao C., Sun J. (2023). Melatonin for premenstrual syndrome: A potential remedy but not ready. Front. Endocrinol..

[20] Shao S., Zhao H., Lu Z., Lei X., Zhang Y. (2021). Circadian Rhythms Within the Female HPG Axis: From Physiology to Etiology. Endocrinology.

[21] Vigil P., Meléndez J., Soto H., Petkovic G., Bernal Y.A., Molina S. (2022). Chronic Stress and Ovulatory Dysfunction: Implications in Times of COVID-19. Front. Glob. Women’s Health.

[22] Baker F.C., Lee K.A. (2022). Menstrual cycle effects on sleep. Sleep Med. Clin..

[23] Chung Y., Kim H., Koh D.-H., Park J.-H., Yoon S. (2021). Relationship Between Shift Intensity and Insomnia Among Hospital Nurses in Korea: A Cross-sectional Study. J. Prev. Med. Public Health.

[24] Rapkin A.J., Winer S.A. (2009). Premenstrual syndrome and premenstrual dysphoric disorder: Quality of life and burden of illness. Expert Rev. Pharm. Outcomes Res..

